# Preconditioning of Microglia by α-Synuclein Strongly Affects the Response Induced by Toll-like Receptor (TLR) Stimulation

**DOI:** 10.1371/journal.pone.0079160

**Published:** 2013-11-13

**Authors:** Cintia Roodveldt, Adahir Labrador-Garrido, Elena Gonzalez-Rey, Christian C. Lachaud, Tim Guilliams, Rafael Fernandez-Montesinos, Alicia Benitez-Rondan, Gema Robledo, Abdelkrim Hmadcha, Mario Delgado, Christopher M. Dobson, David Pozo

**Affiliations:** 1 CABIMER, Andalusian Center for Molecular Biology and Regenerative Medicine, Seville, Spain; 2 Department of Medical Biochemistry, Molecular Biology and Immunology, School of Medicine, University of Seville, Seville, Spain; 3 Institute of Parasitology and Biomedicine ‘Lopez-Neyra’, CSIC, Granada, Spain; 4 Department of Chemistry, University of Cambridge, Cambridge, United Kingdom; University of Cambridge, United Kingdom

## Abstract

In recent years, it has become accepted that α-synuclein (αSyn) has a key role in the microglia-mediated neuroinflammation, which accompanies the development of Parkinson’s disease and other related disorders, such as Dementia with Lewy Bodies and Alzheimer’s disease. Nevertheless, the cellular and molecular mechanisms underlying its pathological actions, especially in the sporadic forms of the diseases, are not completely understood. Intriguingly, several epidemiological and animal model studies have revealed a link between certain microbial infections and the onset or progression of sporadic forms of these neurodegenerative disorders. In this work, we have characterized the effect of toll-like receptor (TLR) stimulation on primary murine microglial cultures and analysed the impact of priming cells with extracellular wild-type (Wt) αSyn on the subsequent TLR stimulation of cells with a set of TLR ligands. By assaying key interleukins and chemokines we report that specific stimuli, in particular Pam3Csk4 (Pam3) and single-stranded RNA40 (ssRNA), can differentially affect the TLR2/1- and TLR7-mediated responses of microglia when pre-conditioned with αSyn by augmenting IL-6, MCP-1/CCL2 or IP-10/CXCL10 secretion levels. Furthermore, we report a skewing of αSyn-primed microglia stimulated with ssRNA (TLR7) or Pam3 (TLR2/1) towards intermediate but at the same time differential, M1/M2 phenotypes. Finally, we show that the levels and intracellular location of activated caspase-3 protein change significantly in αSyn-primed microglia after stimulation with these particular TLR agonists. Overall, we report a remarkable impact of non-aggregated αSyn pre-sensitization of microglia on TLR-mediated immunity, a phenomenon that could contribute to triggering the onset of sporadic α-synuclein-related neuropathologies.

## Introduction

The synucleinopathies are a group of pathologies increasingly affecting the population over 65 years old, comprising various progressive, neurodegenerative disorders including Parkinson’s disease (PD), dementia with Lewy bodies (DLB) and multiple system atrophy (MSA) [1]. Despite the particular characteristics regarding the type of cells and brain areas affected, these disorders have in common the accumulation of α-synuclein (αSyn) insoluble aggregates as the main pathological feature [2]. Moreover, αSyn has also been identified as a component of amyloid brain tissues in AD patients [3]. PD, the most prevalent of these pathologies, is characterized pathologically by the presence of intraneuronal inclusions highly enriched in αSyn (known as Lewy bodies) in the substantia nigra (SN) of the brain [4-6], and the loss of dopaminergic neurons [7]. Three missense mutations of αSyn, A30P, E46K, and A53T, as well as multiple copies of the wild-type (Wt) gene, are linked to rare, early onset PD cases [7]. Even though αSyn is now recognized as a key player in the pathogenesis of PD and other αSyn-related disorders, the cellular and molecular events underlying its pathological actions are not understood in detail. Moreover, the underlying mechanisms driving the development of sporadic PD and other synucleinopathies and which constitute the vast majority clinical cases, remain largely unknown [2,8]. 

Accumulated evidence shows that inflammation is involved in the pathogenesis of a number of neurodegenerative diseases including PD, AD, and multiple sclerosis (MS), among others [9]. Indeed, it is now well established that the onset and progression of PD is accompanied by a robust inflammatory response essentially mediated by activated microglia in affected areas of the brain, which is thought to precede neuronal degeneration [10-12]. Moreover, several *in vitro* studies have revealed the ability of exogenous αSyn, especially the PD-linked mutational variants and the oligomeric forms of αSyn, to induce such a response by stimulated microglia [13-15]. This finding is particularly important as the presence of αSyn has been detected in extracellular biological fluids including human cerebrospinal fluid (CSF) [16-19] and also shown to be secreted from neuronal cells [19-21]. Importantly, imbalances in Wt extracellular αSyn levels –both aggregated and monomeric- have been detected in CSF from patients with PD, AD, DLB and certain forms of prion diseases [22-26]. Even though it is unclear to what extent such variations in CSF mirrors the concentrations within key brain regions and whether they arise at the very initial stages of disease, they indicate that non-aggregated extracellular αSyn within the local environment could be highly relevant for pathogenesis.

Intriguingly, several epidemiological and animal studies have revealed a link between certain bacterial, viral and parasitic infections and the development or progression of sporadic PD [27-33] and AD [34-36]. Nevertheless, the mechanism by which infectious agents or inflammatory stimuli could exacerbate or modulate these microglial phenotypes is not well understood.

Activation of microglia and inflammation in the context of PD, amyotrophic lateral sclerosis (ALS), and AD is currently thought to involve the toll-like receptors (TLRs) [37-39], a group of transmembrane proteins known to detect invading pathogens [40]. TLRs have been reported to be up-regulated in α-synucleinopathy-derived brain tissue from mouse and human subjects [41,42], and have been proposed to mediate different pathways leading to either neuroprotective or neurotoxic phenotypes [42-45]. Although it has recently been shown that treatment of cells with aggregated αSyn is able to alter the TLRs gene expression in microglial cells [46], there are as yet no reported studies on the cellular mechanisms modelling a pre-oligomeric stage of the disease that could recapitulate the setting of neuroinflammation and neurodegeneration. This fact highlights the importance of studying the impact of extracellular αSyn on the innate immune response following TLR stimulation. In this work, by characterizing the effect of a set of TLR agonists on primary microglia cultures, we report a remarkable impact of Wt αSyn priming of cells on TLR-mediated immunity that might reflect a causal link between certain infections and the initiation of sporadic neurodegenerative disease.

## Materials and Methods

### TLR ligands

TLR ligands were purchased from InvivoGen (San Diego, USA) and prepared according to the manufacturer’s recommendations. The TLR ligands were: bacterial lipopolysacharyde (LPS), type B CpG oligonucleotide ODN 1688 (CpG), low molecular weight polyinosine-polycytidylic acid (poly(I:C-LMW)) (PolyI:C), lipoteichoic acid from *B. subtilis* (LTA), synthetic bacterial lipoprotein Pam3CSK4 (Pam3), peptidoglycan from *B. subtilis* (PGN), imiquimod (Imiq), and ssRNA40/LyoVec (ssRNA).

### α-synuclein protein overexpression, purification and characterization

Human Wt αSyn was overexpressed in *E. coli* BL21(DE3) cells using plasmid pT7-7 and purified as described previously [14]. The purity and monomeric state of the αSyn preparation (>95%) was assessed by 15% SDS-PAGE, mass spectrometry analysis, and 4-10% native PAGE (Lonza, Basel, Switzerland), as previously described [14]. The preparation and characterization of soluble α-synuclein oligomers was carried as reported previously [47] and purified oligomeric fractions were stored at 4 °C for up to 24 hrs. Endotoxin levels in the protein preparations were measured by the ToxiSensor Chromogenic LAL Assay Kit (GenScript, Piscataway, USA), and values obtained were <1 EU/mg protein in all cases. The protein concentration of non-aggregated and oligomeric αSyn was determined by means of Micro BCA Reagent Kit (Pierce, Rockford, IL, USA).

### Preparation and characterization of primary microglial cell cultures

Mixed glial cultures were prepared from cerebral cortices of 1-3 day-old C57BL/6 male mice (University of Seville Animal Core Facility, Seville, Spain), and the microglial fraction was isolated, according to previously described methods [14]. Purified microglial cells were characterized by immunocytochemistry as cells of the haematopoietic lineage on the basis of their expression of the pan haematopoietic marker CD45 and of the monocyte/macrophage markers CD11b, F4/80 and CD68, as described elsewhere [14]. Additionally, the absence of glial fibrillary acidic protein (GFAP)-positive astrocytes in purified microglial cell cultures was also assessed according to a previously reported method [14].

### Treatment of microglial cell cultures

Stimulation of microglial cultures in 12-well plates was performed by replacing the medium by adding 1 mL Wt αSyn (or medium alone in the case of the following controls: ‘untreated microglia’ and ‘TLR ligand alone’ controls), at a final concentration of 1 µg/mL (equivalent to 70 nM) diluted in complete DMEM-F12 medium. After a 6-hour incubation at 37 °C, 110 µL aliquot of TLR ligand solutions (at 10x higher concentration) in complete DMEM-F12 medium was added to each well containing either αSyn-primed, or non-primed cells. In the case of some controls, 110 µL of medium alone was instead added to the wells. The final concentrations of the ligands being tested correspond to conditions reported previously [48], and were: LPS, 1 µg/mL; CpG, 1 µg/mL; Poly (I:C), 50 µg/mL; LTA, 10 µg/mLl; Pam3, 1 µg/mL; PGN, 10 µg/mL; Imiq; 1 µg/mL; and ssRNA, 0.25 µg/mL. Cell culture samples with αSyn-primed cells but with no subsequent TLR-ligand stimulation, as well as cells with no addition of αSyn or TLR ligands, were used as controls. Cells were then incubated for a further period of 18 hrs. In addition, some controls were prepared in parallel by incubating cells for 24 hrs with 1 µg/mL A30P αSyn or oligomeric Wt αSyn. In all cases, after incubation for a total of 24 hrs, the supernatants were harvested and the cells were frozen and stored at -80 °C. 

### Cytokine release measurements

After treatment and incubation of cells for a total of 24 hrs as explained in the previous subsection, culture supernatants were harvested and centrifuged at 700 g for 5 min. The supernatants from treated cultures were recovered and stored at -80 °C before measurement of cytokine levels. IL-6, TNFα, IL-1ß, IL-10, IL-13 and IL17 levels were assayed using the mouse IL-6/IL-10 BD OptEIA ELISA set (BD Biosciences, Franklin Lakes, NJ, USA), the murine IL-13 ELISA Development Kit (Peprotech, London, UK), and the mouse IL-17 DuoSet® ELISA Development System (R&D Systems, Minneapolis, USA), according to the manufacturer's protocols. Chemokine levels in the culture supernatants were determined by a specific sandwich ELISA by using capture/biotinylated detection antibodies obtained from Peprotech (London, UK) according to the manufacturer’s recommendations.

### Determination of TLR gene expression

Expression levels of the genes for TLRs 1, 2, 3, 4 and 7, and for hypoxanthine-phophoribosyltransferase (HPRT), were determined by using a two-step quantitative real-time PCR (qRT-PCR) method. Total RNA from treated microglial cells was extracted using the Tripure Isolation Reagent (Roche, Basel, Switzerland) according to the manufacturer's protocol. RNA (1 µg) was reverse-transcribed by using the Quantitect Reverse Transcription kit (Qiagen GmbH, Hilden, Germany) according to the manufacturer's protocol. qRT-PCR was performed with SensiFAST™ SYBR Lo-ROX Kit (Bioline, London, UK) on an ABI Prism 7500 Real Time PCR System. Primer pairs were designed to anneal in different exons, and were: HPRT_For: 5’-GTAATGATCAGTCAACGGGGGAC-3’, HPRT_Rev: 5’-CCAGCAAGCTTGCAACCTTAACCA-3’; primers for TLR genes were purchased from Sigma (Sigma-Aldrich, St. Louis, USA). TLR4_For: 5’-ACCAGGAAGCTTGAATCCCT-3’; TLR4_Rev: 5’-TCCAGCCACTGAAGTTCTGA-3’; TLR7_For: 5’-TCAAAGGCTCTGCGAGT-3’; TLR7_Rev: 5’-AGTCAGAGATAGGCCAGGA-3’. TLR1, TLR2 and TLR3 primers were purchased from Qiagen (Hilden, Germany). Multiple transcripts were analyzed simultaneously for 40 cycles using an optimized qRT-PCR thermal profile. Changes in gene expression were determined using the ∆Ct value taking *hprt* as endogenous control. The ∆∆Ct values were calculated by subtracting ∆Ct values of non-primed samples followed by TLR stimulation (‘TLR ligand’) from samples treated with αSyn-priming followed by TLR stimulation (‘Wt+TLR ligand’).

### Phagocytosis assays

Fluoresbrite^TM^ carboxylate microspheres of 0.75 µm diameter (2.64 % Solid-Latex; Polysciences Inc, Warrington, USA) were used as fluorescein-conjugated tracker microparticles for measuring the phagocytosis capacity of differentially activated microglial cells. 1 hr before starting the phagocytosis assay, FITC-labelled microspheres (1.08 x 10^11^ particles/mL) were mixed at a ratio of 1 µL microspheres: 20 µL FBS for 1 hr at 37 °C into inactivated FBS (BioWhittaker, Verviers, Belgium) and incubated for a further 1 hr at 37 °C in order to opsonise fully the carboxylate groups. The mixtures of microspheres and FBS were then resuspended in fresh DMEM-F12 medium (BioWhittaker, Verviers, Belgium), with L-glutamine and P/S antibiotics supplements to obtain normal 10% FBS- supplemented media containing 5.4 x 10^8^ microspheres/mL. After removal of 500 µL of supernantant from the αSyn-stimulated microglial cell cultures for cytokine release analyses, a volume of 150 µL of resuspended microspheres was added to the remaining 600 µL in each well to obtain a final concentration of 1x10^8^ particles/mL. Particles were then homogenously distributed throughout the well by gentle movement of the plate and incubated for 1 hr at 37 °C. Medium containing non-phagocytosed microspheres was then removed and the cells were washed with PBS prior to fixation with 4% *p*-formaldehyde in PBS for 30 min at 4 °C. A volume of 1 mL of PBS containing the nuclear fluorescent dye bisBenzimide H 33342 tri-hydrochloride (Hoechst 33342; 1 µg/mL) was then added to the cells, and the plates were stored at 4 °C for a minimum of 24 hrs until being analyzed. For this purpose, an Olympus IX71 fluorescence microscope equipped with the digital image processing softwares DPController and DPManager (Olympus Europa, Hamburg, Germany), was used. For each sample, the phagocytic capacity of microglial cells was determined by analysing fluorescent images of phagocytosed FITC-labelled microspheres and Hoechst-stained nuclei from four randomly chosen fields (each containing ~85 cells). For each random field, the total numbers of spheres and nuclei were determined using the Granularity application of the digital imaging analysis software Metamorph (MDS Analytical Technologies, Toronto, Canada), and the number of spheres per nucleus, as an indicator of the phagocytic capacity, was calculated for every field analysed. The values shown correspond to the mean from two or three independent experiments (N=2 or 3), each one containing duplicate samples.

### Determination of Arg1/iNOS gene expression

Total RNA from treated microglial cells was extracted and reverse-transcribed as described before in this section. The primers used were as follows: for mouse arginase-1/Arg1 (band size: 264 bp): Forward: 5´-CAGAAGAATGGAAGAGTCAG-3´; Reverse: 5´-CAGATATGCAGGGAGTCACC-3´, for mouse iNOS (band size: 373 bp): Forward: 5´-GCCTCATGCCATTGAGTTCATCAACC-3´; Reverse: 5´-GAGCTGTGAATTCCAGAGCCTGAAG-3´, and for mouse actin (band size: 165 bp): Forward: 5´-TGTTACCAACTGGGACGACA-3´; Reverse: 5´-GGGGTGTTGAAGGTCTCAAA-3´. DNA Marker: Fermentas* phiX174 DNA/BsuR I (Hae III) Marker, 9.

The positive controls for the Arg and iNOS PCR assays (PCR+) were bone marrow derived macrophages either non stimulated or stimulated 24 hrs with IL-4 (10 ng/mL), respectively. Macrophages were isolated from bone marrow from Balb/c mice and cultured as follows: bone marrow cells (0.4 x 10^6^/mL) were cultured in DMEM (2 mM L-glutamine, 100 units/mL penicillin/streptomycin and 20% heat-inactivated FCS, all from Gibco/Invitrogen) containing 20 ng/mL M-CSF (Peprotech) for 7-8 days. Differentiated macrophages were detached by incubating the plates with 2 mM EDTA/PBS at 37 °C for 10 min. Cell preparations typically consisted of >95% CD11b^+^CD11c^-^ macrophages. Bone marrow derived macrophages were plated at 8 x 10^5^ cells/well in 6-well plates. After 4 hrs of adherence, macrophages were washed with PBS and stimulated with IL-4 (BD Bioscience; 10 ng/mL) for 24 hrs.

Final concentrations in the PCR reaction mixture were: cDNA template: 60 ng; dNTPs: 0.2 mM; primers: 0.4 µM; MgCl_2_: 2 mM; Taq polimerase (Biotools): 0.625 units/reaction, in a total volume of 25 µL. PCR conditions were as follows: for Arg1: 94°C-5 min; 94°C-30 sec, 56°C-30 sec, 72°C-30 sec (30 cycles); 72°C-7 min; 4°C-o/n. For iNOS: 94°C- 35 sec; 62°C-2 min; 72°C-2 min (35 cycles); 72°C-7 min; 4°C-o/n. For actin: 94°C-5 min; 94°C-30 sec, 60°C-30 sec, 72°C-30 sec (30 cycles); 72°C-7 min; 4°C-o/n.

### Determination of cleaved caspase-3 levels by ELISA

For detecting activated caspase-3 protein levels by ELISA, the Human/Mouse Cleaved Caspase-3 (Asp175)-DuoSet ELISA kit (R&D Systems, Abingdom, UK) was used. For this, total protein was extracted from treated microglial cells in culture with ‘lysis buffer’ according to the manufacturer’s instructions, and quantified with the BCA Protein Assay Reagent Kit (Thermo Fisher Scientific Inc., Rockford, USA).

### Determination of cleaved caspase-3 levels by immunofluorescence

For immunofluorescence (IF) analysis, highly pure microglial cell cultures were obtained as described above (in the ‘Preparation and characterization of primary microglial cell cultures’ section), but in this case cultures were prepared on a hydrophilic µ-Dish (Ibidi GmbH, Germany). 3-4 days after isolation, microglial cells were treated as described before (in the ‘Preconditioning of microglial cell cultures with αSyn and stimulation with TLR ligands’ section). For the immunolabelling step, treated microglial cultures were thereafter fixed in cold PBS containing 4% p-formaldehyde for 15 min at 4 °C and then washed in PBS prior to being permeabilized and blocked in PBS containing 3% BSA (Sigma-Aldrich, St. Louis, USA) and 0.5 % Triton X-100 (Sigma-Aldrich, St. Louis, USA) for 1 hr at 4 °C. Cells were incubated o/n at 4 °C with cleaved caspase-3 (Asp175) antibody (Cell Signalling Technology Inc., Danvers, USA) at a 1/200 dilution in PBS containing 3% BSA and 0.5% Triton X-100. After 3 washes with PBS, cells were incubated for 1 hr at room temperature in the dark with a donkey anti-rabbit IgG-AlexaFluor 594 secondary antibody (Invitrogen, Paisley, UK) at a 1/800 dilution in the same buffer. After three washes with PBS, the nuclei were counterstained by incubating cells with PBS containing 1 µg/mL Hoechst 33342 (Sigma-Aldrich, St. Louis, USA) for 1 hour at 4 °C and examined under the fluorescence microscope. Fluorescence images were captured with an Olympus IX71inverted fluorescence microscope equipped with digital image processing softwares DPController and DPManager (Olympus. www.olympus.co.uk). Fluorescence images were taken at x20 magnification from randomly chosen fields. A rigorous comparative evaluation of cleaved caspase-3 immunoexpression was achieved by taking fluorescence images with the same exposure time. Fluorescence images (cleaved caspase-3 marker and nuclear Hoechst 33342 stainings) taken for different microglial cell cultures were finally merged at the same ratio with the use of DPManager software. Phase-contrast images were also obtained from the same cultures, at x10 magnification. For quantitative analysis of cleaved caspase-3 levels by IF, the imaging software MetaMorph Offline (version 7.5.1.0, MDS Analytical technologies, USA), was used. Analysis was performed by using the ratio of the intensity values of Alexa 594 nm (RF) and Hoechst 33342 (BF) above background (areas lacking cells), and the data were expressed as arbitrary units (AU) and exported automatically from to Microsoft Excel program trough a summary log. For measuring the relative cleaved caspase-3 levels per cell, the total specific RF/BF (red fluorescence/ blue fluorescence) ratio was calculated. 

### Data analysis

All values are expressed as the mean ± S.E.M. Statistical significance was evaluated by the Student’s t-test using SPSS Statistics 19.0 (IBM Company, Chicago, USA). Statistically significant differences in relative cytokine release levels, phagocytosis and TLR expression [(Wt+TLR ligand): (TLR ligand) fold-changes] were calculated with the t-Student test between two sets of results by comparing the values under conditions of αSyn-preconditioning followed by TLR stimulation (Wt+TLR ligand) relative to conditions in the absence of αSyn preconditioning and TLR stimulation (TLR ligand). Each set of results originated from several independent experiments (N=3-7). In addition, in the cases of statistically significant changes, the absolute values were also compared by applying a two-way Student’s t-test for each (Wt+TLR ligand) sample condition to those resulting from stimulation of cells with αSyn alone (‘Wt αSyn’) or untreated cells (‘Control’). For quantitative analysis of cleaved caspase-3 levels by IF, three images from random fields (N=3), each containing 80-90 cells, were used to calculate the mean RF/BF value and SEM for each sample condition. For quantitative ELISA assays, results originated from three independent experiments (N=3). Statistically significant differences of results from IF and ELISA assays were calculated by applying the Student’s t test in relation to the values obtained with the corresponding TLR ligand in the absence of αSyn-preconditioning and with Wt αSyn alone. In all cases, statistically significant differences between the two sets of results were those with *p*<0.05.

### Ethics Statement

All animals were handled in strict accordance with good animal practice as defined by the relevant national/EU guidelines and The CEA-CABIMER Experimental Animal Committee, and all animal work was approved by the appropriate committee (file CEA-2010-14).

## Results

### Priming of microglia with Wt αSyn primarily affects the TLR2 and TLR7-mediated immune response

Given that the underlying mechanisms initiating and accompanying the development of sporadic synucleinopathies remain essentially unknown, we decided to carry out our studies with Wt αSyn protein. Although various types of cells have been identified as a source of cytokines in the central nervous system (CNS), microglia appear to be a principal source of pro-inflammatory and immune regulatory cytokines [9]. In order to explore the possible impact of αSyn as a priming factor in the microglial immune response following pathogen invasion, we challenged Wt αSyn-primed microglial cultures with a set of TLR agonists, namely Pam3Csk4 lipopeptide (Pam3; TLR2/1), peptidoglycan from *B. subtilis* (PGN; TLR2), lipoteichoic acid from *B. subtilis* (LTA; TLR2), Poly(I:C)-LMW ―a synthetic analog of dsRNA- (PolyI:C; TLR3), bacterial lipopolysaccharide (LPS; TLR4), imiquimod ―a small synthetic antiviral molecule- (Imiq; TLR7), ssRNA40 oligonucleotide complexed with LyoVec (ssRNA; TLR7), and type B CpG oligonucleotide (CpG; TLR9). We omitted the study of TLR5 stimulation based on previous reports of its absence in mouse microglia [44,49], and on our own observations of a lack of effect with the TLR5 ligand flagellin as measured by secreted levels of proinflammatory cytokines (data not shown).

After incubation with 1 µg/mL (equivalent to ca. 70 nM) of non-aggregated Wt αSyn for 6 hrs (or mock solution in the case of non-preconditioned samples and untreated controls), the cell cultures were incubated for a further 18 hrs with standard concentrations of different TLR agonists, or otherwise treated with medium alone to serve as controls. The αSyn working concentration was chosen considering the typical range from previous reports and especially based on our earlier work in which a similar experimental setup has been used [14]. After incubation of cells for a total of 24 hrs, the supernatants were recovered for later analysis of their interleukin/chemokine contents. Given that the aggregated αSyn as well as the A30P αSyn variant have been previously shown to exert a stronger pro-inflammatory effect on microglia [14,50], control samples were prepared by incubating cells for 24 hrs with 1 µg/mL A30P αSyn variant or 1 µg/mL oligomeric αSyn species.

A set of key interleukins, namely pro-inflammatory IL-6, immunoregulatory IL-10, anti-inflammatory IL-13, and autoimmunity-related IL-17, were assayed by ELISA (Table **1**). Our results show that, at the concentrations used, all eight TLR ligands, as well as A30P and oligomeric αSyn, produced higher IL-6 secretion when added alone to microglial cells, relative to untreated controls (Table **1**). In addition, stimulation with LPS and Poly I:C caused an increase in IL-10 and IL-13 levels, respectively, while oligomeric αSyn appeared to cause a reduction in IL-13 secreted levels (Table **1**). 

**Table 1:** Values of secreted interleukins in primary microglia 24 hrs after treatment.

	**IL-6 (pg/mL)**	**IL-10 (pg/mL)**	**IL-13 (pg/mL)**	**IL-17 (pg/mL)**
**Control**	64.4 ± 10.3	120 ± 34,4	44.1 ± 12.6	8.6 ± 1.1
**Wt αSyn**	198 ± 89.8	20.8 ± 12.9	72.3 ± 21.6	9.2 ± 0.8
**A30P αSyn**	1880 ± 604	150.2 ± 83.2	59.0 ± 19.0	11.6 ± 4.4
**Oligomeric Wt αSyn^a^**	481 ± 130	N/A	33.1 ± 10.8	N/A
**LPS**	7719 ± 2231	253 ± 37.5	61.6 ± 8.1	9.8 ± 1.4
**CpG**	1542 ± 296	182 ± 45.4	65.6 ± 12.5	9.0 ± 1.2
**Pam3**	1604 ± 283	111.4 ± 57.3	47.8 ± 7.4	11.0 ± 2.0
**Imiq**	1041 ± 235	66.5 ± 18.6	48.1 ± 6.67	8.9 ± 1.2
**ssRNA**	256 ± 40	68.5 ± 26.1	66.8 ± 20.6	9.9 ± 1.4
**PGN**	1037 ± 379	63 ± 25.6	61.0 ± 10.8	7.9 ± 0.9
**LTA**	1731± 297	118.5 ± 20.1	66.7 ± 17.2	7.9 ± 1.2
**Poly I:C**	1641 ± 541	64 ± 23.9	178.0 ± 34.2	7.9 ± 1.0

**Values of secreted interleukin levels in primary microglia 24 hrs after treatment**. Following treatment of cells with αSyn or with TLR agonists at the concentrations described in the Materials and Methods section, cell culture supernatants were harvested after incubation for 24 hrs, and cytokine levels were assayed by ELISA. Values correspond to the mean of six independent experiments (N=6) each containing duplicate samples and error corresponds to SEM, except for (a , in which the values shown are the mean of two independent experiments (N=2) each containing four replicas and the error corresponds to SD.

**Figure 1:**
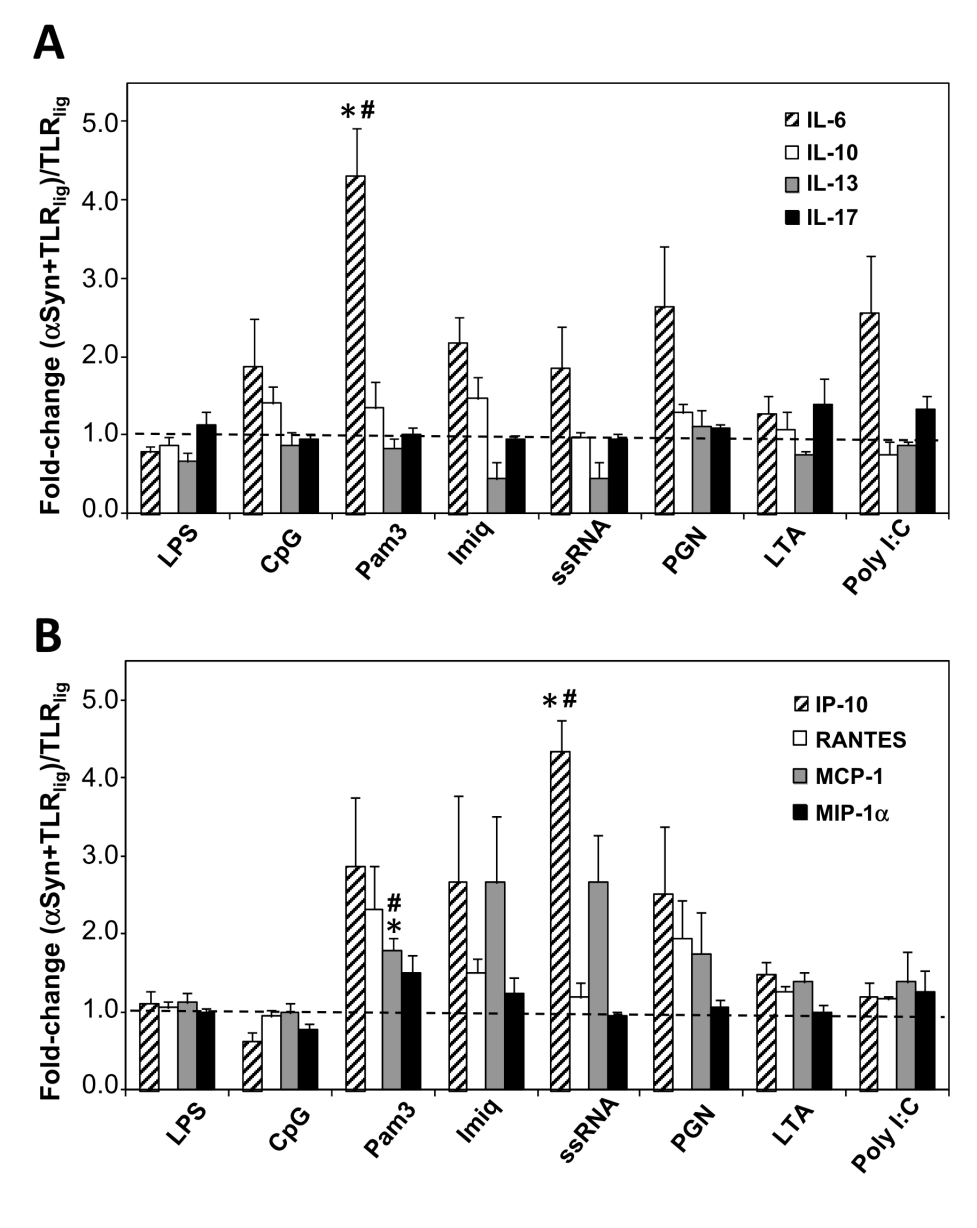
Impact of Wt αSyn-priming on microglial cytokine release after TLR stimulation. After treating the microglial cells either with Wt αSyn at 1 µg/mL (‘priming’ or pre-conditioning) or with ‘mock’ solution (no pre-conditioning) for 6 hrs, the TLR agonists were added to their specified final concentrations (see Materials and Methods), and incubated for further 18 hrs at 37 °C. The culture supernatants were harvested and used to measure (**A**) the levels of the interleukins IL-6, IL-10, IL-13, and IL-17, or (**B**) of the chemokines IP-10/CXCL10, RANTES/CCL5, MCP-1/CCL2, by ELISA. Values are the fold-change calculated as the signal ratio of αSyn-primed, TLR-stimulated cells (‘αSyn+TLR ligand’) relative to non-primed, TLR-stimulated cells (‘TLR ligand’). The results shown (mean ± SEM) are the average of several independent experiments (IL-6: N=5-7; IL-10: N=4-6; IL-13: N=3; IL-17: N=2-3; IP-10, RANTES, MCP-1, and MIP-1α: N=3-5), each containing duplicate samples. Statistically significant differences (* *p*<0.05) were calculated by applying the Student t test between the two sets of results, for all the TLR ligands tested. (#) denotes a result that is significantly different from that obtained after treatment of cells with Wt αSyn alone (p<0.05).

We then sought to assess the impact of αSyn-preconditioning of microglia on the interleukin secretion profile (Figure **1A**). A general increase trend in TNFα secreted levels could be observed for the primed cells after TLR stimulation, in particular with (TLR7) ssRNA, (TLR2/1 Pam3) and (TLR2) PGN (Figure **S1**). This effect was not seen for IL-1ß, as only (TLR3) Poly I:C produced an increase trend in its secretion levels under αSyn priming conditions (Figure **S1**). On the other hand, a significant 4-fold increase in IL-6 release levels was observed for the Wt αSyn-primed cells after stimulation with Pam3 (TLR2/1) (*p*=0.024), relative to the corresponding controls with the TLR agonists in the absence of priming with αSyn. Of particular note is that neither IL-10 nor IL-17 levels displayed changes in Wt αSyn-primed cells for any of the tested TLR ligands. Interestingly, even though they did not reach statistical significance, moderate fold-change reductions of IL-13 anti-inflammatory cytokine levels were observed in the cases of stimulation of primed microglia with the TLR7 agonists Imiq and ssRNA40 (Figure **1A**)**.**

We then analysed the secretion profile of treated cells for a set of key chemokines, namely IP-10/CXCL10, RANTES/CCL5, MCP-1/CCL2, and MIP-1α/CCL3 (Table **2**). Virtually all eight TLR ligands tested, as well as A30P and aggregated αSyn, produced an increase in chemokine secretion for all four chemokines tested when added to cells, relative to untreated controls (Table **2**). Remarkably, preconditioning of cells with αSyn resulted in a 4.5-fold increase in IP-10 levels upon stimulation with ssRNA (TLR7) (*p*=0.015) (Figure **1B**). Furthermore, a 2-fold increase in the MCP-1 level was measured for the case of stimulation with Pam3 (TLR2/1) (*p*=0.024), and a similar, although not statistically significant, trend was seen with Imiq and ssRNA (TLR7) (Figure **1B**). On the other hand, small and non significant increases were observed in relative levels of RANTES with LTA (TLR2) or MIP-1α with Pam3, relative levels. Importantly, all of the increases described above that were statistically significant proved to be independent of the sole addition of αSyn (in all cases *p* values were <0.05), implying that these alterations arise from a complex combination of αSyn-priming and subsequent TLR-stimulation effects on microglia.

**Table 2:** Values of secreted chemokines in primary microglia 24 hrs after treatment.

	**IP-10 (pg/mL)**	**RANTES (pg/mL)**	**MCP-1 (ng/mL)**	**MIP-1α (pg/mL)**
**Control**	16.9 ± 12.9	315 ± 88	1.8 0.4	11.8 ± 7.2
**Wt αSyn**	47.2 ± 27.5	505 ± 255	3.8 ± 1.1	4.9 ± 3.3
**A30P αSyn**	2290 ± 656	1887 ± 395	11.8 ± 5.9	696 ± 276
**Oligomeric Wt αSyn^a^**	870 ± 341	N/A	6.8 ± 1.7	N/A
**LPS**	3693 ± 303	3967 ± 767	27.0 ± 4.6	1832 ± 289
**CpG**	1503 ± 557	2183 ± 323	14.8 ± 2.8	1276 ± 285
**Pam3**	610 ± 273	1289 ± 225	15.5 ± 3.9	973 ± 75
**Imiq**	436 ± 192	1891± 224	18.0 ± 5.0	1277 ± 171
**ssRNA**	209 ± 114	609 ± 147	4.5 ± 1.1	176 ± 105
**PGN**	549 ± 229	722 ± 275	9.9 ± 4.8	291 ± 92
**LTA**	2454 ± 490	2970 ± 620	18.4 ± 3.3	1479 ± 287
**Poly I:C**	3116 ± 460	3679 ± 725	20.8 ± 2.4	943 ± 178

**Values of secreted chemokine levels in primary microglia 24 hrs after treatment**. Following treatment of cells with αSyn or with TLR agonists at the concentrations described in the Materials and Methods section, cell culture supernatants were harvested after incubation for 24 hrs, and chemokine levels were assayed by ELISA. Values correspond to the mean of six independent experiments (N=6) each containing duplicate samples and error corresponds to SEM, except for (a , in which the values shown are the mean of two independent experiments (N=2) each containing four replicas and the error corresponds to SD.

### αSyn-preconditioning of microglia alters TLR expression when stimulated with Imiq, PGN and Poly I:C

**Figure 2:**
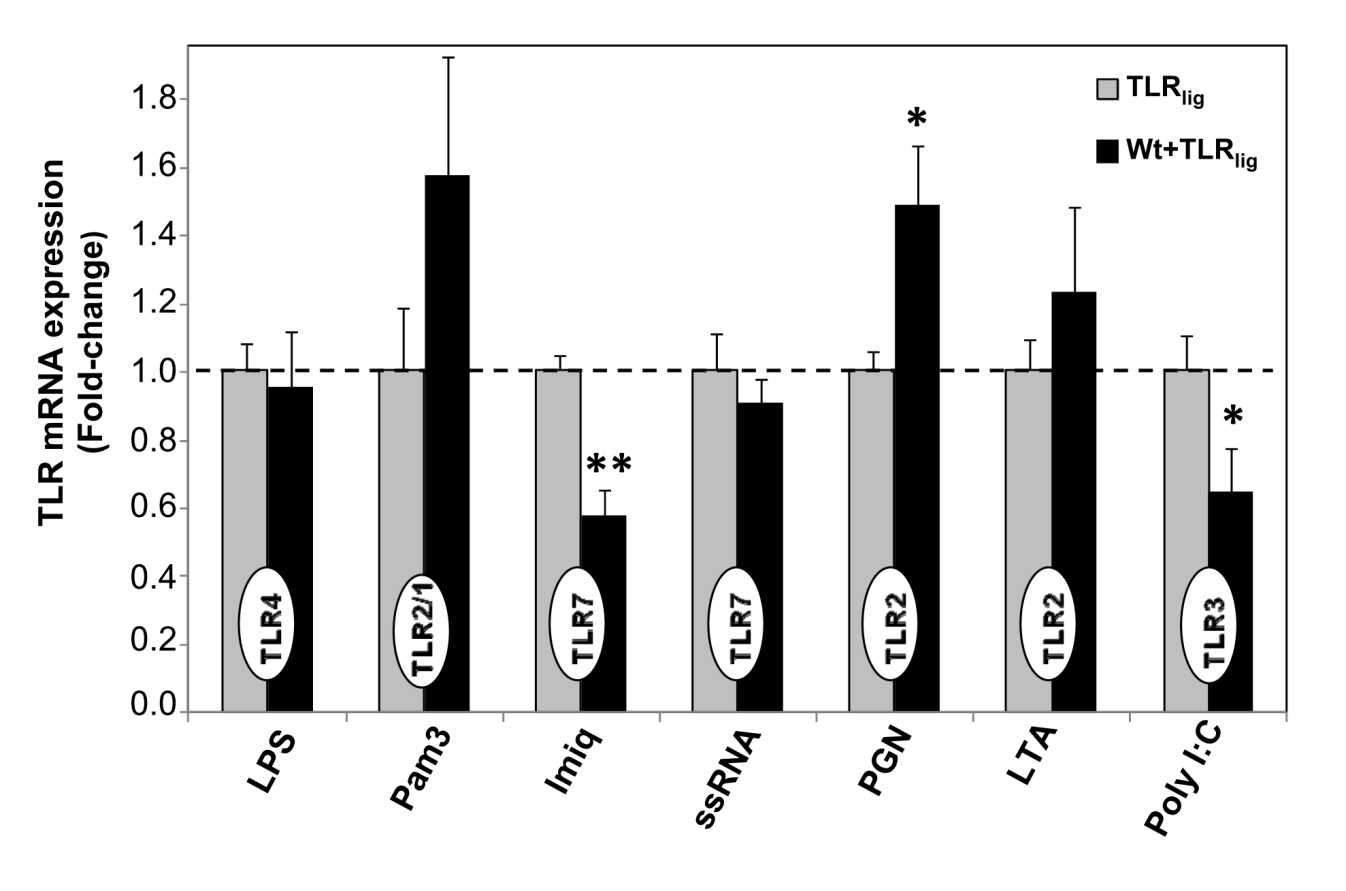
Comparison of TLR gene expression levels of Wt αSyn-primed vs. **non-primed microgia, after TLR stimulation**. After treating cells as before (see legend to Figure 1), cells were lysed and the total RNA was extracted. Relative TLR gene expression levels were then examined by qRT-PCR and the *hprt* gene was used as the internal control to calculate ∆Ct values. The ∆∆Ct values were calculated by subtracting ∆Ct values of non-primed samples upon TLR stimulation (‘TLR ligand’) from ∆Ct values of samples treated with αSyn-priming upon TLR stimulation (‘Wt+TLR ligand’), to give the fold-change in TLR expression in cells with ‘αSyn+TLR ligand’ relative to ‘TLR ligand’ treatments. In all cases the TLR gene analysed (indicated inside bars) corresponded to the TLR agonist used in that particular sample. Fold-changes represent the average of three independent experiments (N=3), each one performed with duplicate samples. Bars correspond to SEM. Statistically significant differences (* *p*<0.05) were calculated by applying the Student t test between the two sets of results, for all the TLR ligands tested.

Given the substantial changes observed in the cytokine secretion profiles as a consequence of the preconditioning process, we sought to investigate whether or not such alterations could result from changes in TLR expression levels. Therefore, we measured the mRNA levels of TLRs 2, 3, 4 and 7 in the samples after treatment as described above, and compared the change in TLR expression in αSyn-preconditioned vs. non-preconditioned, cells. Because stimulation with CpG (TLR9) ligand showed essentially no change trends in either cytokine release levels as a consequence of priming with αSyn, we omitted further studies with this ligand and continued our characterization with the seven remaining TLR agonists tested (Figure **2**). On the one hand, significant suppression of TLR7 and TLR3 expression levels were detected in αSyn-preconditioned cells upon stimulation with Imiq (*p*=0.002) and Poly I:C (*p*=0.030) ligands, respectively. On the other hand, a modest increase in TLR2 expression was measured αSyn-preconditioned cells stimulated with PGN (*p*=0.031) (Figure **2**). However, even though moderate increases in TLR expression were observed for samples stimulated with Pam3 (TLR 2/1), and LTA (TLR2), only a 50% increment in the case of PGN (TLR2) reached statistical significance (*p*=0.031), while no changes were measured for the cases of LPS and ssRNA (Figure **2**). These results suggest that targeting receptor-ligand interactions rather than TLR expression could provide a better rationale and strategy for the management of α-Syn-related pathologies. 

### Impact of αSyn priming on microglial phagocytic capacity upon TLR stimulation

**Figure 3:**
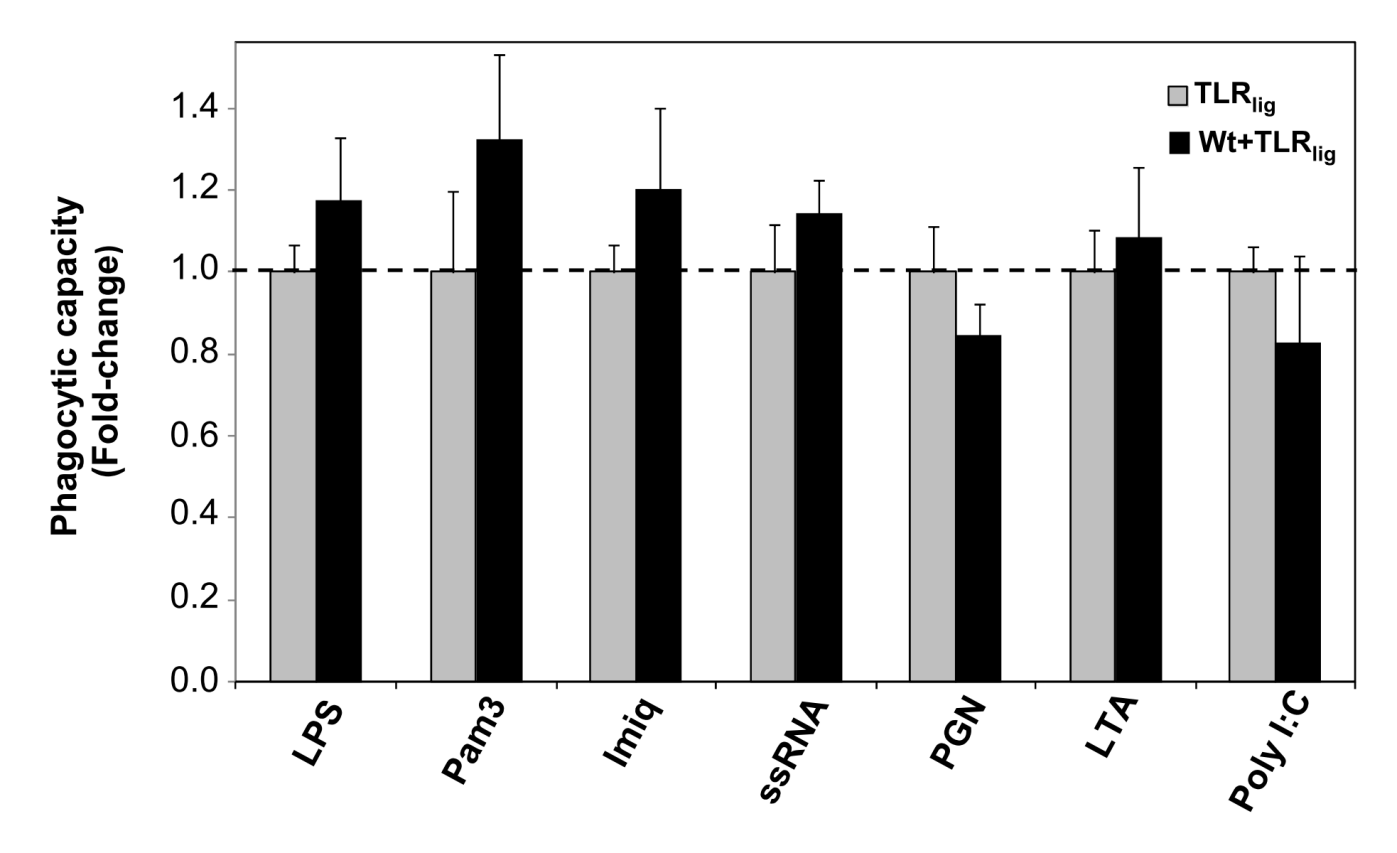
Relative phagocytic capacity of WtαSyn-primed *vs*. non-primed microglia after TLR stimulation. After treatment of the primary microglial cell cultures and incubation for a total of 24 hrs as described in the legend to Figure 1, cells were incubated with fluorescent microspheres for 1 hr. After fixing the cells, phagocytosis was assessed by fluorescence microscopy and calculation of the number of spheres/cell as described in the Methods section. Four images were analysed for each sample in each independent experiment. The ‘relative phagocytic capacity’ corresponds to the ratio (fold-change) of the number of spheres/cell of αSyn-primed cells followed by TLR stimulation (‘αSyn+TLR ligand’) relative to the number of spheres/cell of non-primed, TLR-stimulated cells (‘TLR ligand’). The values shown are an average of three (for ssRNA and PGN) or four (for all the others) independent experiments (N=3 or 4), and error bars represent the SEM. Statistically significant differences (* *p*<0.05) were calculated by applying the Student t test between the two sets of results, for all the TLR ligands tested. (#) denotes a result that is significantly different from that obtained after treatment of cells with Wt αSyn alone (p<0.05).

Recently, it was reported that, in contrast to the aggregated form, monomeric αSyn enhances the microglial phagocytic capacity [51]. Indeed, our previous observation that non-aggregated Wt αSyn promotes a moderate but significant increase in microglial phagocytosis [14] is consistent with this finding. Therefore, we sought to test whether or not the changes previously observed in the secretion of specific cytokines for αSyn-primed microglia, following stimulation by certain TLR ligands, could be accompanied by alterations in the relative phagocytic capacity of microglia that could be possibly linked to the pathogenesis of the synucleinopathies. For this purpose, we used fluorescein-conjugated tracker microparticles to test the phagocytic capacity of αSyn-primed vs. non-primed microglia, following stimulation with certain TLR ligands (Figure **3**). Despite the fact that a certain trend towards moderate increases in microglial phagocytosis was noticed in the cases of stimulation with (TLR2/1) Pam3 of Wt-primed microglia (~30%), the differences did not reach statistical significance (Figure **3**) and therefore we can conclude that the contribution of αSyn preconditioning in the neurodegenerative process or immune imbalance after TLR triggering, within the microglial environment, is independent of phagocytosis.

### α-*Syn* preconditioning of microglia followed by stimulation with TLR ligands leads to differential profiles of cell polarization

**Figure 4:**
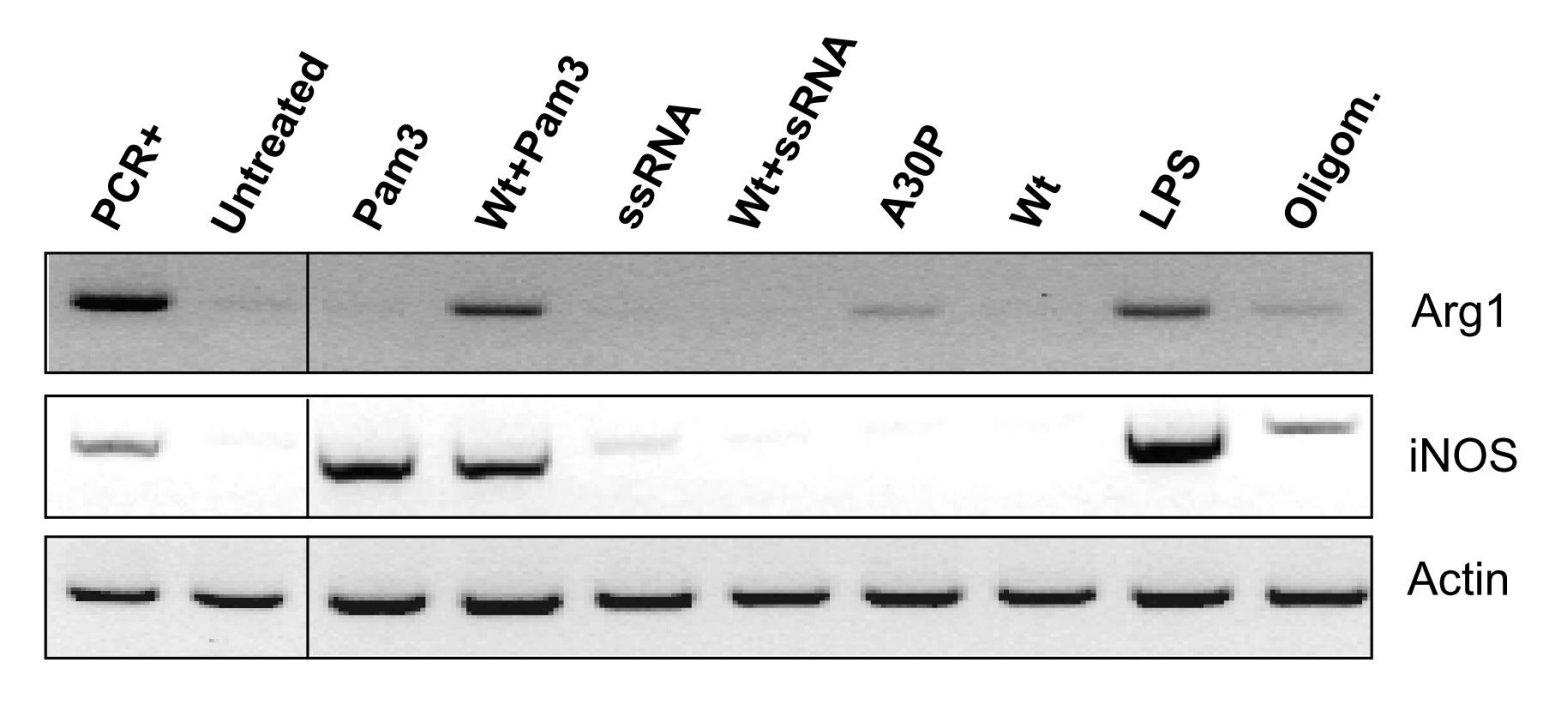
Arg1 and iNOS PCR gene expression assays of αSyn-preconditioned microglia stimulated with Pam3 or ssRNA. Primary microglial cells were either treated with Wt αSyn (Wt), A30P αSyn (A30P), or oligomeric Wt αSyn (oligomers) at 1 µg/mL, or with culture medium alone, for 6 hrs at 37 °C. Subsequently, the TLR agonists or medium alone were added accordingly, and cells were incubated for a further 18 hrs as described before. After treatment, RNA was extracted and reverse transcribed for PCR analysis of arginase-1 (Arg1) and iNOS gene expression. Actin expression was used as a reference and the positive controls for the PCR assays (PCR+) were from bone marrow-derived macrophages stimulated (for Arg1) or not (for iNOS) for 24 hrs with IL-4 (10 ng/mL).

In recent years, it has become clear that, as a result of exposure to microenvironmental signals, microglial cells can undergo alternative polarized activation modes. The two extreme phenotypes of macrophages are defined as M1 (the classical, proinflammatory macrophages) and M2 (the ‘alternatively activated’/resolving anti-inflammatory cells); however, a full spectrum of activation states which share some overlapping properties with those of the poles, are currently thought to exist [52]. To gain further insight into the effect on cell phenotype of αSyn-primed microglia after stimulation with Pam3 and ssRNA TLR ligands, we assayed the gene expression of standard M1 and M2 phenotypic markers iNOS and Arg1, respectively [53]. As can be observed (Figure **4**), treatments with either Pam3 or ssRNA alone produced an iNOS^+^/Arg1^-^ phenotype which, in combination with their cytokine release profiles (higher secreted levels of IL-6 or IP-10 and lower levels IL-10) (Table **1**, Figure **1**), indicate varying degrees of polarization towards an M1-like state. 

Interestingly, treatment of αSyn-primed cells with ssRNA (as well as with Wt αSyn alone) produced an iNOS^-^/Arg1^-^ (double negative) phenotype, while stimulation of cells with A30P αSyn induced the expression of the Arg1 marker. Remarkably, treatment of αSyn-preconditioned microglia with Pam3 agonist, just like exposing cells to oligomeric αSyn, produced an iNOS^+^/Arg1^+^ (double positive) intermediate phenotype (Figure **4**). This result was also the case for treatment with LPS, which is consistent with reports of ‘M2 skewing’ and the Arg1^+^ phenotype of microglia produced by administration of LPS *in vitro* [54] and *in vivo* [55,56]. The iNOS^+^/Arg1^+^ phenotype observed in these particular samples, together with the increase in secreted IL-6, TNFα, IP-10 and MCP-1 levels (Tables **1** and **2**) and with an essentially unaltered phagocytic capacity (Figure **2**), suggests a skewing towards an M1/M2 intermediate or mixed microglial phenotype. 

### αSyn-preconditioning alters activated caspase-3 levels in microglia following stimulation with Pam3 and ssRNA

**Figure 5:**
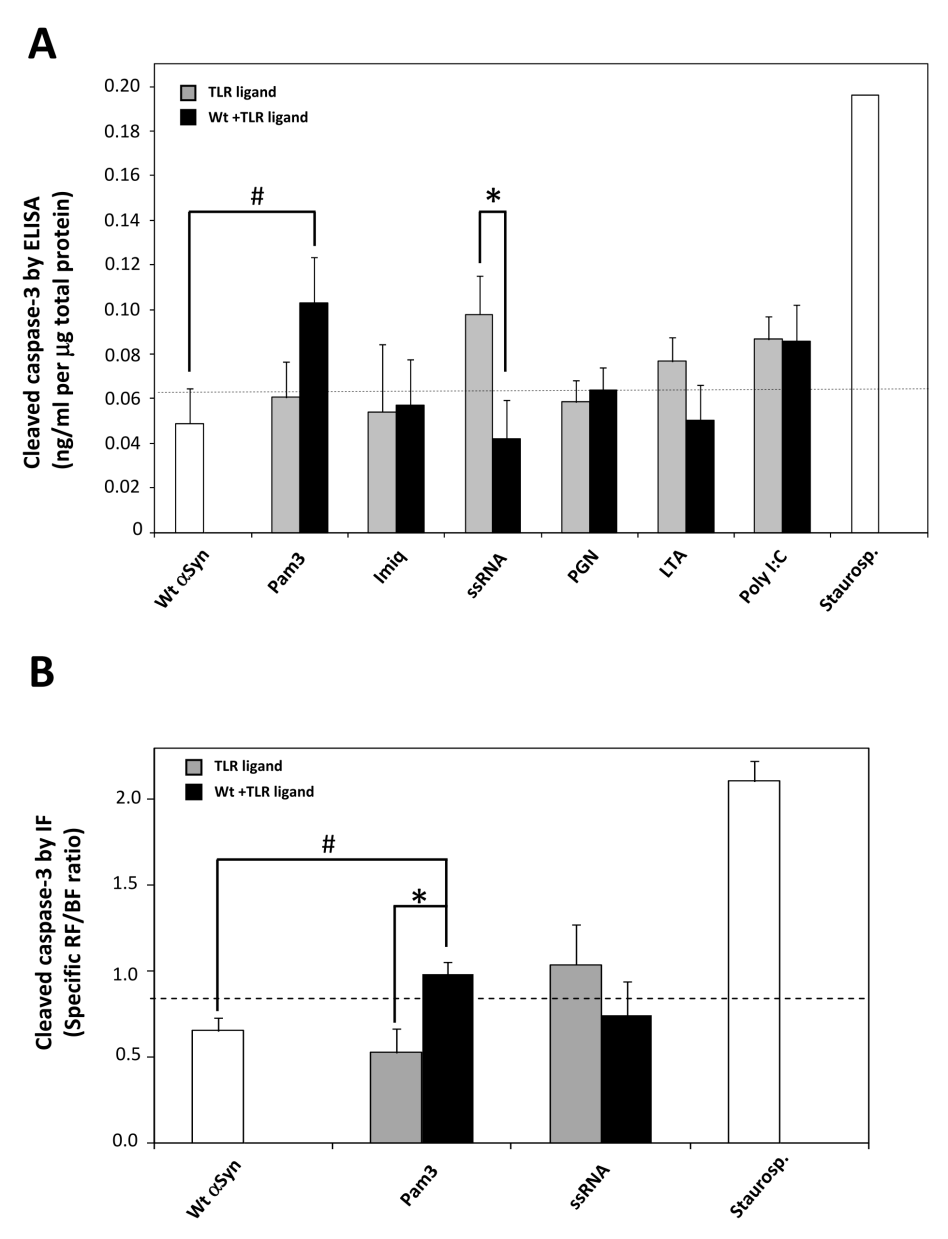
Quantitation of activated caspase-3 levels in treated microglial cells, by ELISA and immunofluorescence. (**A**) After treatment of the primary microglial cell cultures and incubation for a total of 24 hrs as described in the legend to Figure 1, cells were lysed and tested by a specific ELISA assay for cleaved caspase-3 levels quantitation. The results shown (ng/mL cleaved casp-3 per µg of total protein) correspond to the mean of four independent experiments (N=4), each one performed with duplicate samples. Bars correspond to SEM. A discontinuous line represents the mean value obtained for untreated cells. Statistically significant differences were calculated by applying the Student’s t test in relation to the values obtained with the corresponding TLR ligand in the absence of αSyn-preconditioning (* *p*<0.05) and with Wt aSyn alone (# *p*<0.05). Treatment with staurosporine from *Streptomyces* sp. (5 µM) for 6 hrs was used as a positive control. (**B**) Cells were cultured in appropriate culture plates and treated as explained above (see legend to Figure 1) for subsequent labelling of cleaved caspase-3 and nuclear Hoechst 33342 staining for IF analysis, as described in the Methods Section. Samples were analyzed under the fluorescence microscope and three images from random fields containing ca. 80-90 cells each, were recorded, and analyzed for fluorescence quantification. The total specific red fluorescence (RF) and blue fluoresce (BF) were measured and the RF/BF ratio was used as a quantitation method and is represented in this figure. The results shown (RF/BF ratio) correspond to the mean of three images analysed (N=3) within one representative experiment, and bars correspond to SEM. A discontinuous line represents the mean value obtained for images from untreated cells. Statistically significant differences were calculated by applying the Student’s t test in relation to the values obtained with the corresponding TLR ligand in the absence of αSyn-preconditioning (* *p*<0.05) and with Wt αSyn alone (# *p*<0.05). Treatment with staurosporine from *Streptomyces* sp. (5 µM) for 6 hrs was used as a positive control.

**Figure 6:**
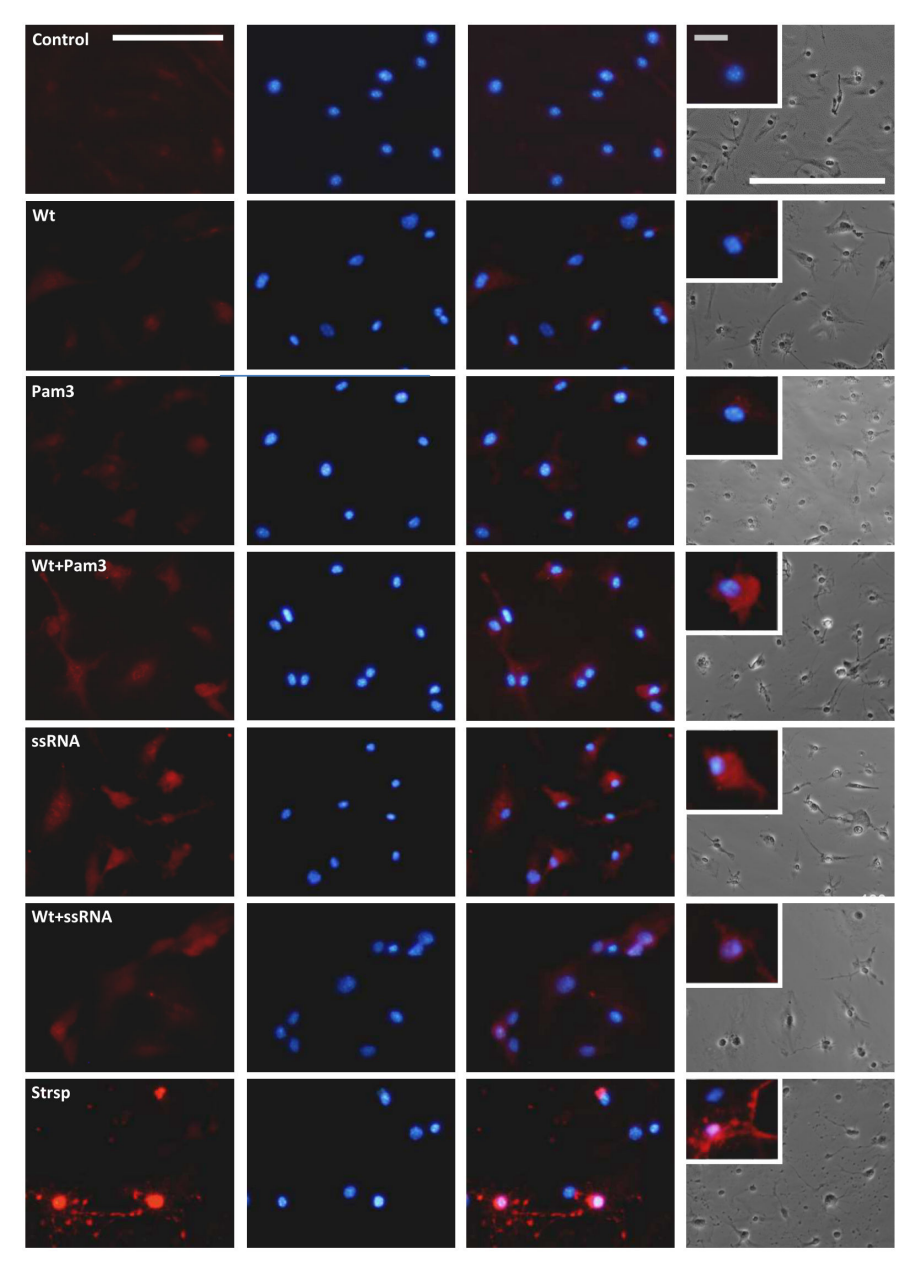
Immunofluorescence analysis of cleaved caspase-3 levels in treated microglial cells. Representative images taken from immunolabeled primary microglial cells treated as described in the legend to Figure 1, with or without preconditioning by Wt αSyn. The TLR ligands tested were Pam3 and ssRNA40 at the concentrations described before (see Materials and Methods). Cells treated with 5 µM staurosporine (Strsp) from *Streptomyces* sp. for 6 hrs served as a positive control. Specific anti-(Asp175) cleaved caspase-3 primary antibodies and Alexa Fluor 594 secondary antibodies were used to visualize activated caspase-3 (first column), and nuclei were counterstained with Hoescht (second column). Merged images are shown in the third column and phase-contrast images of the same cultures are shown in the right column. White scale bars: 50 µm; grey scale bar (inset): 5 µm.

Even though there is a strong link established between the activation of microglia and the progression of PD, the molecular pathways linking microglia-mediated neuroinflammation and neurodegeneration have been elusive. Activated caspase-3 has been observed in the SN of patients with PD [57-59], specifically in microglia within the SN of human subjects suffering from PD and AD [60]. Furthermore, caspase-3 was recently shown to have a key role in the regulation of microglia activation and neurotoxicity [60]. Therefore, we sought to compare the expression of activated caspase-3 in primary microglia produced by the different treatments (Figure **5A**). Except for an increase produced by stimulation of cells with (TLR7) ssRNA and (TLR3) Poly I:C, the basal cleaved caspase-3 levels were not significantly altered by treatment with the TLR ligands or the Wt α-Syn alone. However, higher cleaved caspase-3 levels were indeed observed for Wt αSyn-preconditioned microglia upon stimulation with (TLR2/1) Pam3. Remarkably, the observed increase induced by (TLR7) ssRNA alone was strongly suppressed by preconditioning of cells with Wt αSyn (Figure **5A**). Furthermore, these alterations were also observed by immunofluorescence (IF) analyses of primary microglial cultures that were similarly treated, by using specific antibodies against cleaved caspase-3 (Figure **5B** and Figure **6**). As observed for the untreated cells (‘control’), treatment of microglia with Wt αSyn or (TLR2/1) Pam3 alone barely produced detectable levels of activated caspase-3. However, preconditioning of cells with Wt αSyn and subsequent stimulation with Pam3 produced a general increase in fluorescence (ca. 2-fold, p=0.031), located primarily in the cytosol (Figure **6**). Finally, stimulation of cells with (TLR7) ssRNA ligand alone produced an increase in cleaved caspase-3 protein, which localized essentially in the cytosol. In agreement with the results obtained by ELISA (Figure **5A**), preconditioning of cells with Wt αSyn was found to suppress such an ssRNA-induced increase, by IF analysis. Moreover, this lower level of activated caspase-3 was observed mainly to localize in the cell nuclei (Figure **6**).

## Discussion

Despite much progress done in recent years, the underlying mechanisms that trigger the onset of the sporadic form of several neurodegenerative diseases including AD, PD, DLB, and ALS, remain to be elucidated. Given that they all have in common a strong inflammatory response mediated by activated microglia, the existence of additional factors that could potentially exacerbate such neuroinflammatory process is currently thought to be pivotal. Indeed, it has been proposed that microglia in the neurodegenerated brain are somehow ‘primed’, and signals from systemic infection or inflammation trigger an enhanced response that contributes to disease progression [61]. 

In previous studies, by comparing the effects on primary microglia of Wt αSyn with those produced by the PD-linked αSyn mutants, we and others have observed a strong pro-inflammatory microglial response for the A30P and E46K variants, as compared to Wt αSyn [13,14]. Intriguingly, and for reasons still unknown, the levels of brain extracellular αSyn, including its non-aggregated form, have been found to be largely altered in diagnosed patients for several neurodegenerative disorders including AD, PD, DLB, and the prion disease [22-26].

In the present work, we have addressed the question of whether or not preconditioning with non-aggregated Wt αSyn could possibly affect the innate immune response of microglia under conditions of TLR challenge. This issue is of great relevance as it may provide information on the microglia-mediated functional innate response upon infection at the very initial stages of disease onset. 

Our results show that the impact of αSyn-preconditioning of microglia on the innate immune response following stimulation with TLR ligands largely depends on the nature of the subsequent TLR agonist challenge. Indeed, we observed no significant changes in the cytokine secretion profile for certain TLR ligands tested, including LPS (TLR4). The latter is consistent with previous reports of the response following a challenge with LPS performed both on a transgenic mouse model overexpressing Wt αSyn [10] and after injection of non-aggregated Wt αSyn into the mouse SN [62], suggesting that similar inflammatory reactions were induced by LPS independently of the presence of αSyn. Interestingly however, we found that Wt αSyn-primed microglia can indeed affect the immune response mediated by TLR2/1 and TLR7, either by increasing the secretion of the pro-inflammatory cytokines IL-6 and TNFα, or by lowering the expression of the anti-inflammatory IL-13. Interestingly, IL-13 has been shown to reduce dopaminergic neuronal cell mortality within a normal environment, but to contribute to their loss under oxidative stress conditions [63]. 

Very few studies have so far addressed the involvement of chemokines in PD and other related pathologies. In particular, analysis of functional polymorphisms in the genes encoding interleukins and chemokines, and their links with the age of onset or the overall risk of developing PD, has not resulted in any clear associations [64,65]. In addition, the search for chemokine biomarkers of PD in serum has not so far provided useful candidates for diagnosis [66]. In this sense, our results highlight the possible implications of locally affected chemokine environments in primed microglia as a result of specific infections, that have been involved in the recruitment of reactive lymphocytes and in promoting neuronal cell death [67]. According to our results, Wt αSyn-priming additionally affects the Pam3 (TLR2/1)- and ssRNA (TLR7) -stimulated microglia by increasing the secretion levels of the chemokines MCP-1/CCL2 and IP-10/CXCL10, respectively, which have been found to be elevated in the CSF of AD brains from the very early stages of disease and to be linked to neurodegeneration [68]. Overall, the change observed in the cytokine release profiles of αSyn-preconditioned microglia stimulated with Pam3 and ssRNA resemble that generated by treatment with αSyn oligomers and with the A30P αSyn variant, both linked to PD and known to elicit a strong inflammatory response mediated by microglia activation [14,50]. In addition, the differential cytokine secretion profiles upon stimulation with Pam3 and ssRNA are not mediated by significant changes in TLR expression in the case of αSyn-primed cells.

**Figure 7:**
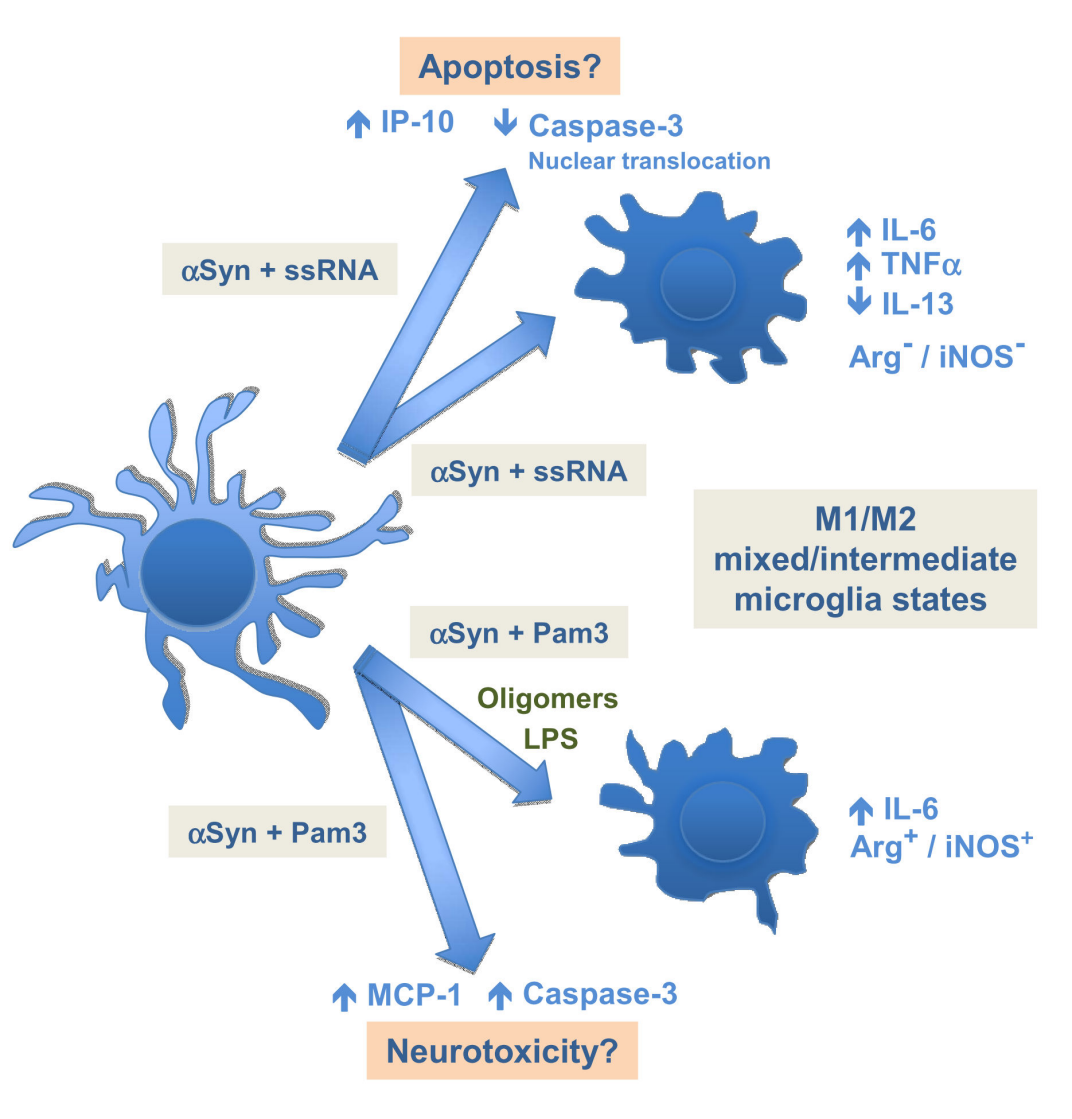
Proposed model of the impact of αSyn-priming and TLR stimulation on microglial phenotype and neuroinflammation. Surveyling microglia undergo polarization towards an M1-like phenotype after exposure to (TLR7) ssRNA and Imiq, and (TLR2/1) Pam3, TLR agonists, characterized by a lack of expression of Arg1, expression of iNOS, and high IL-6 production (see Results). On the one hand, αSyn-preconditioning of microglia and subsequent stimulation with (TLR7) ssRNA (and probably Imiq) produces an Arg1^-^/iNOS^-^ (double negative) mixed or intermediate phenotype, and causes an increase in IP-10 and TNFα secretion, and a reduction of IL-13 levels. In addition, this treatment leads to a reduction in activated caspase-3 levels accompanied with a change in its intracellular location from the cytosol towards the nucleus of the microglial cell. On the other hand, exposure of αSyn-primed microglia to (TLR2/1) Pam3 agonist induces a skewing towards a different M1/M2 mixed or intermediate phenotype, exhibiting an Arg1^+^/iNOS^+^ (double positive) expression pattern, together with higher IL-6 and MCP-1 secretion levels. Remarkably, this phenotype agrees with the one observed for microglia that have been exposed to oligomeric αSyn. In addition, the ‘αSyn + Pam3’ treatment causes increase in activated caspase-3 levels in microglial cells.

It is now accepted that multiple forms of activated microglia exist, and whether the roles that such differential patterns of activation play in the pathobiology of neurodegenerative diseases are beneficial or detrimental, is currently the subject of much debate [52]. Microglial phagocytosis has traditionally been related to steady-state tissue homeostasis by preventing the release of proinflammatory intracellular components from dead or dying cells, and by contributing to the resolution of inflammation [69,70]. More recently, microglial phagocytosis has also been shown to have a role on neuronal death during inflammation triggered by TLR stimulation [71]. Our present results show that there is no clear effect of αSyn-preconditioning on the phagocytic capacity of TLR-stimulated microglia. In addition, assessment of Arg1 and iNOS gene expression, together with analysis of cytokine release, has revealed a skewing towards an M1/M2 mixed or intermediate activation phenotype for αSyn-preconditioned microglia upon stimulation with ssRNA or Pam3 (Figure **7**). It is noteworthy that an intermediate M1/M2 microglial phenotype has been found *in vivo* in AD murine models [69]. However, the double negative or double positive character of these resulting activation phenotypes suggest that they are of a different nature depending on the particular TLR ligand involved. Our results also indicate that the mixed M1/M2-like response elicited by treatment of αSyn-primed microglia with Pam3 is reminiscent of that displayed after treatment with αSyn oligomers, which are thought to be the most inflammatory and toxic forms of αSyn [2,72,73]. This finding is highly relevant as it has been demonstrated recently that the inoculation of aggregated forms of Wt αSyn into mouse brains is sufficient to trigger PD-like neurodegeneration and the development of PD characteristic symptoms [74,75].

In recent years, a link between activated caspase-3 and PD and AD has been put forward; higher activated caspase-3 levels have been detected in the SN of PD patients [57-59] and specifically in microglial cells within the SN of PD and AD human subjects [60]. Furthermore, activated caspase-3 was recently shown to play a key role in the regulation of microglia activation and to correlate positively with neurotoxicity, initially as a result of TLR stimulation [60]. In this context, our findings indicate that αSyn-primed microglia result in increased activated caspase-3 levels after TLR2/1 engagement by Gram (+) bacteria-related Pam3. On the other hand, virus-like ssRNA produces the opposite effect in addition to relocation from the cytosol to the nucleus, which might suggest an activation of the apoptosis pathway [76], and therefore our findings could also be of potential interest for selective manipulation of apoptotic or neurotoxic signalling pathways in a synucleinopathy-prone scenario. 

In summary, our results show that extracellular wild-type αSyn could potentially act as a priming factor for microglia to produce an altered TLR response as compared to the same challenge in the absence of such priming. Moreover, we show that the features of this altered response are highly dependent on the identity of the agonist engaging such TLR-mediated responses (Figure **7**). We propose that this priming effect could be especially relevant in the case of sporadic synucleinopathies and other related disorders with αSyn imbalances since it postulates that specific infections or inflammatory stimuli, even at the pre-oligomeric stage of the αSyn aggregational process, could potentially act as a trigger of an altered microglial response and accelerate the onset of the disease.

## Supporting Information

Figure S1 **Impact of Wt αSyn-priming on microglial TNFα and IL-1ß release after TLR stimulation.** After treating the microglial cells either with Wt αSyn at 1 µg/mL (‘priming’ or pre-conditioning) or with ‘mock’ solution (no pre-conditioning) for 6 hrs, the TLR agonists were added to their specified final concentrations (see Materials and Methods), and incubated for further 18 hrs at 37 °C. The culture supernatants were harvested and used to measure the levels of TNFα and IL-1ß cytokines by ELISA. Values are the fold-change calculated as the signal ratio of αSyn-primed, TLR-stimulated cells (‘αSyn+TLR ligand’) relative to non-primed, TLR-stimulated cells (‘TLR ligand’). The results shown (mean ± SD) are the average from duplicate samples and is representative of three independent experiments for each cytokine measurement. Untreated cells and treatment of cells with Wt αSyn alone were used as controls in both cases.
(TIF)
