# Supplementary figures and images for: Preconditioning of Microglia by α-Synuclein Strongly Affects the Response Induced by Toll-like Receptor (TLR) Stimulation
Source: PLoS One. 2013 Nov 13;8(11):e79160. doi: 10.1371/journal.pone.0079160 (PMC3827304; doi:10.1371/journal.pone.0079160)

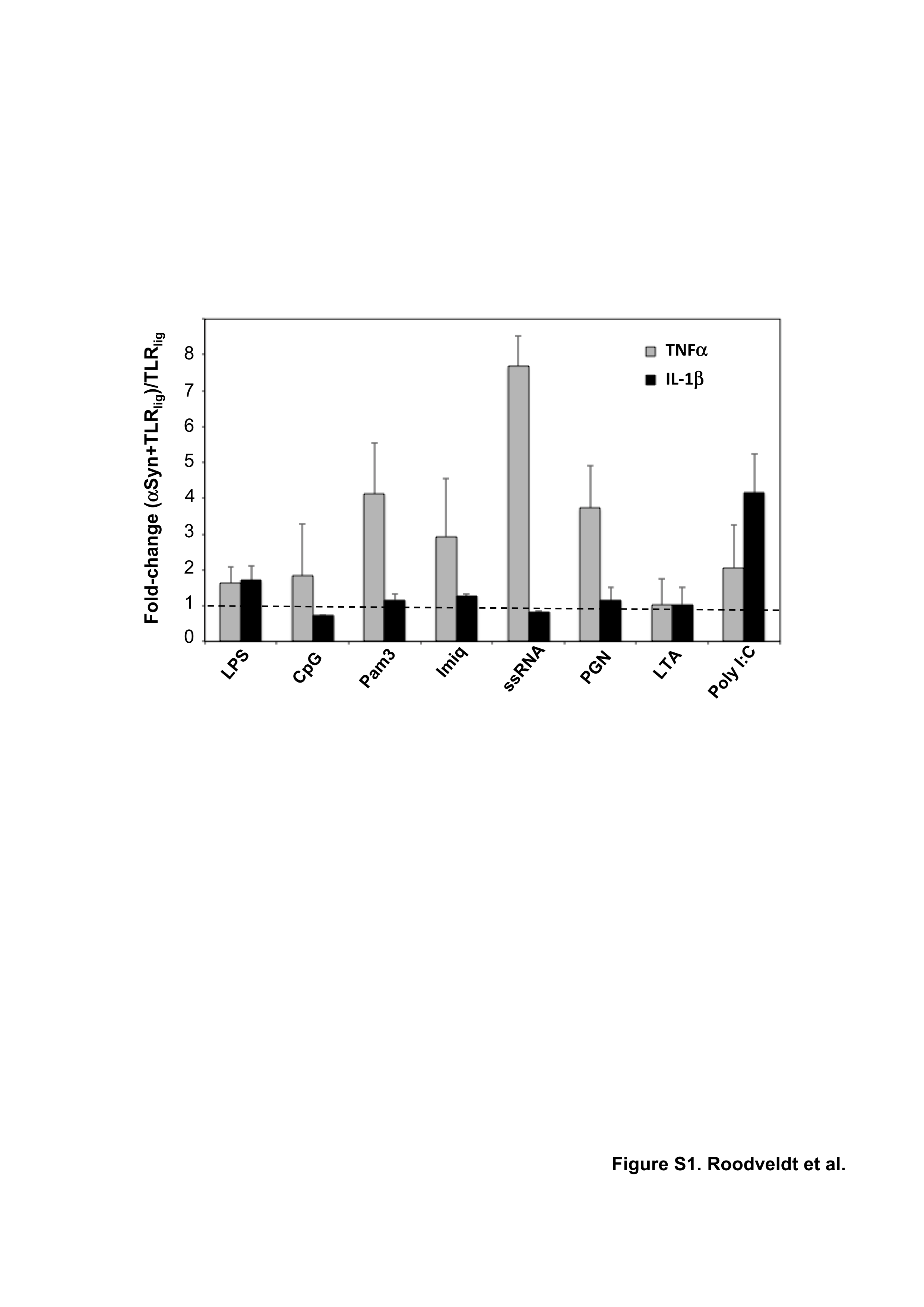

Supplement: Figure S1 — Impact of Wt αSyn-priming on microglial TNFα and IL-1ß release after TLR stimulation. After treating the microglial cells either with Wt αSyn at 1 µg/mL (‘priming’ or pre-conditioning) or with ‘mock’ solution (no pre-conditioning) for 6 hrs, the TLR agonists were added to their specified final concentrations (see Materials and Methods), and incubated for further 18 hrs at 37 °C. The culture supernatants were harvested and used to measure the levels of TNFα and IL-1ß cytokines by ELISA. Values are the fold-change calculated as the signal ratio of αSyn-primed, TLR-stimulated cells (‘αSyn+TLR ligand’) relative to non-primed, TLR-stimulated cells (‘TLR ligand’). The results shown (mean ± SD) are the average from duplicate samples and is representative of three independent experiments for each cytokine measurement. Untreated cells and treatment of cells with Wt αSyn alone were used as controls in both cases. (TIF) [file pone.0079160.s001.tif]
